# Impact of temperature on EC_50_ of ropivacaine in axillary brachial plexus blocks: based on Dixon’s up-and-down method

**DOI:** 10.3389/fmed.2025.1591581

**Published:** 2025-07-14

**Authors:** Alin Wang, Yunhao Shao, Yuling Zheng, Xiaoling Li, Bixin Huang, Ying Mai, Zhongqi Zhang

**Affiliations:** ^1^Department of Anesthesiology, Hospital of Foshan Women and Children, Foshan, China; ^2^Department of Anesthesiology, The Affiliated Shunde Hospital of Jinan University, Foshan, China

**Keywords:** media effective concentration, ropivacaine, temperature, nerve block, up-and-down method

## Abstract

**Objective:**

The efficacy of local anesthetics like ropivacaine in axillary brachial plexus blocks may be influenced by temperature, though its impact on the median effective concentration (EC_50_) remains unclear. This study aimed to determine the EC_50_ of ropivacaine at room temperature (RT, 23°C) and body temperature (BT, 37°C) using the Dixon’s up-and-down method.

**Methods:**

Fifty-nine patients scheduled for upper limb surgery under ultrasound-guided brachial plexus block with ropivacaine were randomly divided into the RT group or BT group, with ropivacaine stored at 23°C for the RT group and 37°C for the BT group. The ropivacaine concentration for each subsequent patient was determined using the up-and-down method. If the sensory nerve block met surgical incision requirements within 30 min, it was classified as “Effective”; otherwise, it was classified as “Ineffective.” For “Effective” cases, the ropivacaine concentration was reduced by 0.05% for the next patient, while for “Ineffective” cases, it was increased by 0.05%. The initial ropivacaine concentration was set at 0.5% for both groups. Probit regression analysis was then used to determine the EC_50_ of ropivacaine. The primary outcome was EC_50_ of ropivacaine, calculated using probit regression. Secondary outcomes included surgical processes indicators and adverse events.

**Results:**

The EC_50_ of ropivacaine was significantly lower in the BT group (0.175, 95% CI: 0.109–0.220%) compared to the RT group (0.243, 95% CI: 0.171–0.289%) (*p* < 0.001). Time to surgical readiness was longer in the BT group (median 25 vs. 13 min, *p* < 0.001), but no differences were observed in adverse events.

**Conclusion:**

Warming ropivacaine to 37°C reduces the EC_50_ of ropivacaine for axillary brachial plexus blocks, suggesting that lower concentrations may achieve effective anesthesia. These findings highlight temperature as a modifiable factor to optimize local anesthetic dosing, potentially minimizing toxicity risks while maintaining efficacy.

**Clinical trial registration:**

https://www.chictr.org.cn/showproj.html?proj=28438 Identifier, ChiCTR1800016721.

## 1 Introduction

The axillary brachial plexus block is commonly used for upper limb surgeries, providing effective anesthesia while minimizing the risks associated with general anesthesia (1–3). Ropivacaine, a long-acting amide-type local anesthetic, is often chosen for these procedures due to its favorable safety profile, including lower cardiotoxicity and neurotoxicity compared to similar agents like bupivacaine (4, 5). Nonetheless, ropivacaine’s clinical effectiveness can vary significantly depending on factors (6), such as concentration and external conditions.

Temperature, an often-overlooked external factor, significantly influences the pharmacodynamics and effectiveness of local anesthetics. The latest research revealed that Warming bupivacaine to 37°C significantly reduces the effective dose required for spinal anesthesia prior to cesarean deliveries (7). Meanwhile, other studies report that heating local anesthetics can cause sensory and motor blockage more quickly (8–11), which means that a lower concentration of ropivacaine may be sufficient for effective nerve blockade. Nevertheless, the impact of temperature on the median effective concentration of ropivacaine remains unclear.

This study aimed to obtain the median effective concentration (EC_50_) of ropivacaine for axillary brachial plexus block at room temperature (RT) and body temperature (BT) using Dixon’s up-and-down method. The findings provide clinical reference for the use of local anesthetics at different temperatures in axillary brachial plexus block.

## 2 Materials and methods

### 2.1 Study design and case screening

A prospective, randomized, double-blind study was conducted from January to September 2024. The study received approval from the Ethics Committee of the Affiliated Shunde Hospital of Jinan University (Approval No. JDSY-LL-2024176) and was registered with the Chinese Clinical Trial Registry (Registration No. ChiCTR1800016721). Informed consent was obtained from all patients.

The inclusion criteria included patients between 18 and 60 years old, with a body mass index (BMI) ranging from 18 to 30 kg/m^2^, an American Society of Anesthesiologists (ASA) physical status classification of I or II, and those scheduled for either elective or emergency upper limb surgery.

The exclusion criteria included patients who declined participation, had a known allergy to ropivacaine, had contraindications to axillary brachial plexus block, or had a recent history of using other analgesic drugs.

### 2.2 Grouping and blinding method

The patients were randomly assigned to the RT or BT groups using computer-generated random numbers. The assignments were sealed in opaque envelopes to ensure allocation concealment. Both the anesthesiologists and patients were blinded to group assignments. The anesthesiologists responsible for the nerve block procedures underwent standardized training to ensure consistency in anesthesia techniques.

### 2.3 Anesthesia procedures

The operating room was kept at a temperature of 23°C. Oxygen was supplied through a face mask at a flow rate of 3–5 L/min. Monitoring included an electrocardiogram (ECG), non-invasive blood pressure (NIBP), and peripheral oxygen saturation (SpO₂), and peripheral venous access was established following standard procedure. All patients received an intravenous infusion of dexmedetomidine for anti-anxiety, starting with a loading dose of 1.0 μg/kg administered over 10 min. This was followed by a continuous infusion at 0.4 μg/kg/h, maintained until the surgery concluded. The method for ultrasound-guided axillary brachial plexus nerve localization was previously described (12). Briefly, after local anesthesia with 1–3 mL of 2% lidocaine, a 50-mm insulated 22-G short-beveled needle (Uniplex® Nanoline®, 22G × 50 mm, Germany) was inserted from the lateral side of the ultrasound probe using an in-plane technique along the short axis. The radial nerve, located posterior-lateral to the artery; the ulnar nerve, positioned superior-medially; the median nerve, located superior-laterally; and the musculocutaneous nerve, situated between the biceps brachii and coracobrachialis fascia, were blocked using a multi-point injection technique. A total of 20 mL of dilute ropivacaine mixed with normal saline was administered, with 5 mL of ropivacaine used for each nerve.

Ropivacaine was diluted with normal saline (pH 4.5–7.0) to achieve different concentrations using the up-and-down method. In the RT group, ropivacaine (100 mg/10 mL, Batch No. NBSD, Manufacturer: AstraZeneca AB, Sweden) was stored in the operating room at ambient temperature (23°C). In the BT group, ropivacaine, diluent (normal saline), and syringes were pre-warmed in a 37°C incubator for at least 30 min before administration. The solutions were mixed in syringes immediately before injection to maintain the target temperature. The initial ropivacaine concentration was set at 0.5% for both groups. If the sensory nerve block met the surgical incision requirements within 30 min, it was classified as “Effective.” Otherwise, it was classified as “Ineffective.” For “Effective” cases, the ropivacaine concentration was decreased by 0.05% for the next patient, while for “Ineffective” cases, it was increased by 0.05%.

For cases of incomplete nerve block, additional anesthesia was administered using intravenous sufentanil at a dosage of 0.1–0.2 μg/kg. If this was still inadequate for surgery, general anesthesia was administered through a laryngeal mask (LMA). In the event of systemic toxicity due to local anesthetics during surgery, established guidelines were followed (13). Puncture-related complications were managed according to the literature (14).

### 2.4 Outcome assessments

The main outcome was the ropivacaine concentration for each patient, determined using the up-and-down method.

The secondary outcomes included: types of injuries/conditions, surgery at 30-min post block (perform surgical incision without additional analgesia within 30 min after the nerve block), the number of patients receiving intravenous sufentanil (cases where additional analgesia was required due to incomplete nerve block), time to surgical readiness (the time interval from the completion of ropivacaine injection to the point when sensory nerve block met surgical incision requirements), the number of patients requiring general anesthesia with LMA (general anesthesia via laryngeal mask airway was necessary due to inadequate nerve block despite intravenous sufentanil administration), and surgery time (the total duration of the surgical procedure), and adverse events, such as local anesthetic toxicity and puncture-related complications.

### 2.5 Statistical analysis

The sample size was determined based on up-and-down method, which required seven crossovers from ‘Effective’ to ‘Ineffective’ for statistical analysis, this method provides stable estimates of EC_50_ with a type I error rate (*α*) of 0.05 and power (1-*β*) exceeding 0.80 for detecting clinically relevant differences in EC_50_ (15–17).

Data were analyzed using SPSS version 20.0 (SPSS Inc., Chicago, IL, USA). Continuous variables were presented as means ± standard deviations (SD) for normally distributed data, and as medians [range] for non-normally distributed data. Categorical data were expressed as frequencies (n, %). The t-test was used to compare normally distributed continuous variables, while the Mann–Whitney U test was applied for non-normally distributed data. Categorical variables were analyzed using either the Chi-square test or Fisher’s exact test, as appropriate. The EC_50_ of ropivacaine were determined through probit regression analysis. Statistical significance was set at a *p*-value of less than 0.05.”Sequential graphs for the up-and-down method and the concentration-effect analysis curve for ropivacaine were plotted using Microsoft Excel.

## 3 Results

### 3.1 Patient characteristics

**Figure 1:**
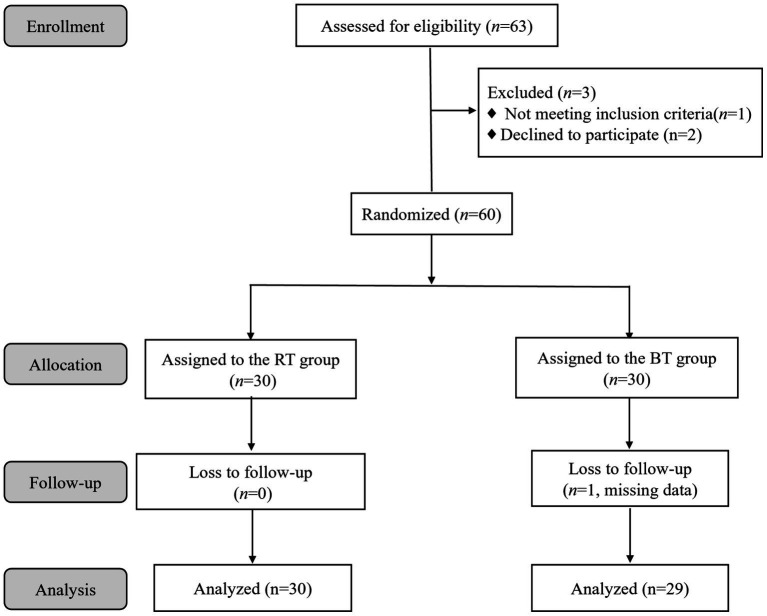
Study flowchart.

A total of 63 patients were assessed for eligibility, with three being excluded. Ultimately, 60 patients were randomly assigned to the RT and BT groups, with 30 patients in each group. One patient in the BT group was lost to follow-up due to missing data, leaving 59 patients who completed the study: 30 in the RT group and 29 in the BT group, as depicted in Figure 1. No significant differences were found between the groups in terms of age, height, weight, BMI, sex, and ASA classification (*p* > 0.05) (Table 1). Additionally, there were no statistically significant differences in the types of surgeries performed between the two groups (*p* > 0.05) (Table 2).

**Table 1:** Patient characteristics.

Characteristics	RT Group	BT Group	*T*/*χ*^2^	*p* value
	*n* = 30	*n* = 29		
Age (years)	39.3 ± 11.4	38.3 ± 11.3	0.336	0.738
Height (cm)	165.9 ± 6.3	164.9 ± 6.5	0.604	0.549
Weight (kg)	63.1 ± 7.8	62.3 ± 9.9	0.356	0.731
BMI (kg/m^2^)	22.9 ± 2.3	22.8 ± 2.9	0.082	0.935
Sex			1.849	0.174
Male	26 (86.7)	21 (72.4)		
Female	4 (13.3)	8 (27.6)		
ASA classification			0.209	0.648
I	11 (36.7)	9 (31)		
II	19 (63.3)	20 (69)		

Data are expressed as means ±SD, *n* (%) or median [range]. ASA, American Society of Anesthesiologists.

**Table 2:** Types of injuries/conditions performed on each group.

Types of injuries/conditions	RT Group	BT Group	*χ*^2^	*p* value
	*n* = 30	*n* = 29		
Forearm fracture	2	4	–	0.424
Hand fracture	14	10	0.472	0.492
Hand deformity	2	3	–	0.671
Hand wound	9	8	0.042	0.838
Wrist fracture	1	2	–	>0.999
Wrist mass	2	2	–	>0.999

Data are expressed as *n*.

### 3.2 Sequential response order between the two groups

**Figure 2:**
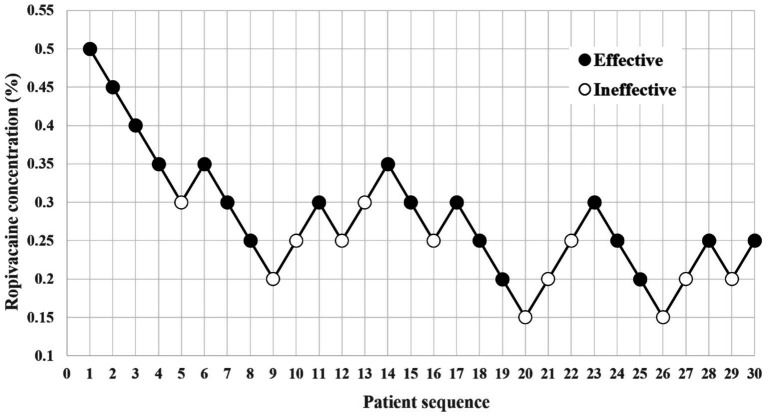
Dixon’s up-and-down method plots for the RT group.

**Figure 3:**
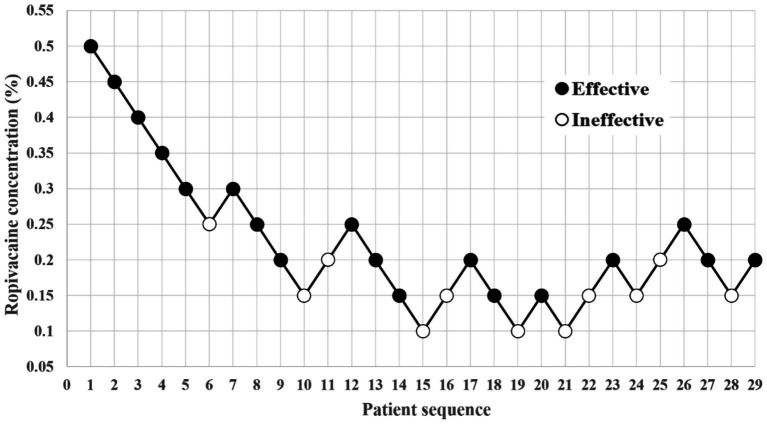
Dixon’s up-and-down method plots for the BT group.

The first patient in each group received a 0.5% ropivacaine. Following seven crossovers using the up-and-down method, the RT group consisted of 30 patients, with 18 classified as ‘Effective’ and 12 as ‘Ineffective’ (Figure 2). In the BT group, 29 patients were included, with 18 classified as ‘Effective’ and 11 as ‘Ineffective’ (Figure 3).

### 3.3 Comparison of indicators related to surgical processes between the two groups

There were no statistically significant differences between the two groups in terms of successful sensory nerve block, surgery at 30-min post block, use of intravenous sufentanil, general anesthesia with LMA, and surgery time (*p* > 0.05). However, the median time to surgical readiness in the RT group was 13 min, while that in the BT group was 25 min, and this difference showed a significant difference (*Z* = 4.107, *p* < 0.001) (Table 3).

**Table 3:** Surgical processes between the two groups.

Item	RT Group	BT Group	*T/Z/χ*^2^	*p* value
	*n* = 30	*n* = 29		
Surgery at 30-min post block	12	12	0.042	>0.999
Intravenous sufentanil	4	8	1.074	0.300
Time to surgical readiness (min)	13[9.5–24]	25[21–35]	4.107	<0.001
General anesthesia with LMA	1	2	-	>0.999
Surgery time (min)	52[40–80]	65[40–100]	0.706	0.561

Data are expressed as *n* or median [range].

### 3.4 Media effective concentration of ropivacaine between the two groups

**Figure 4:**
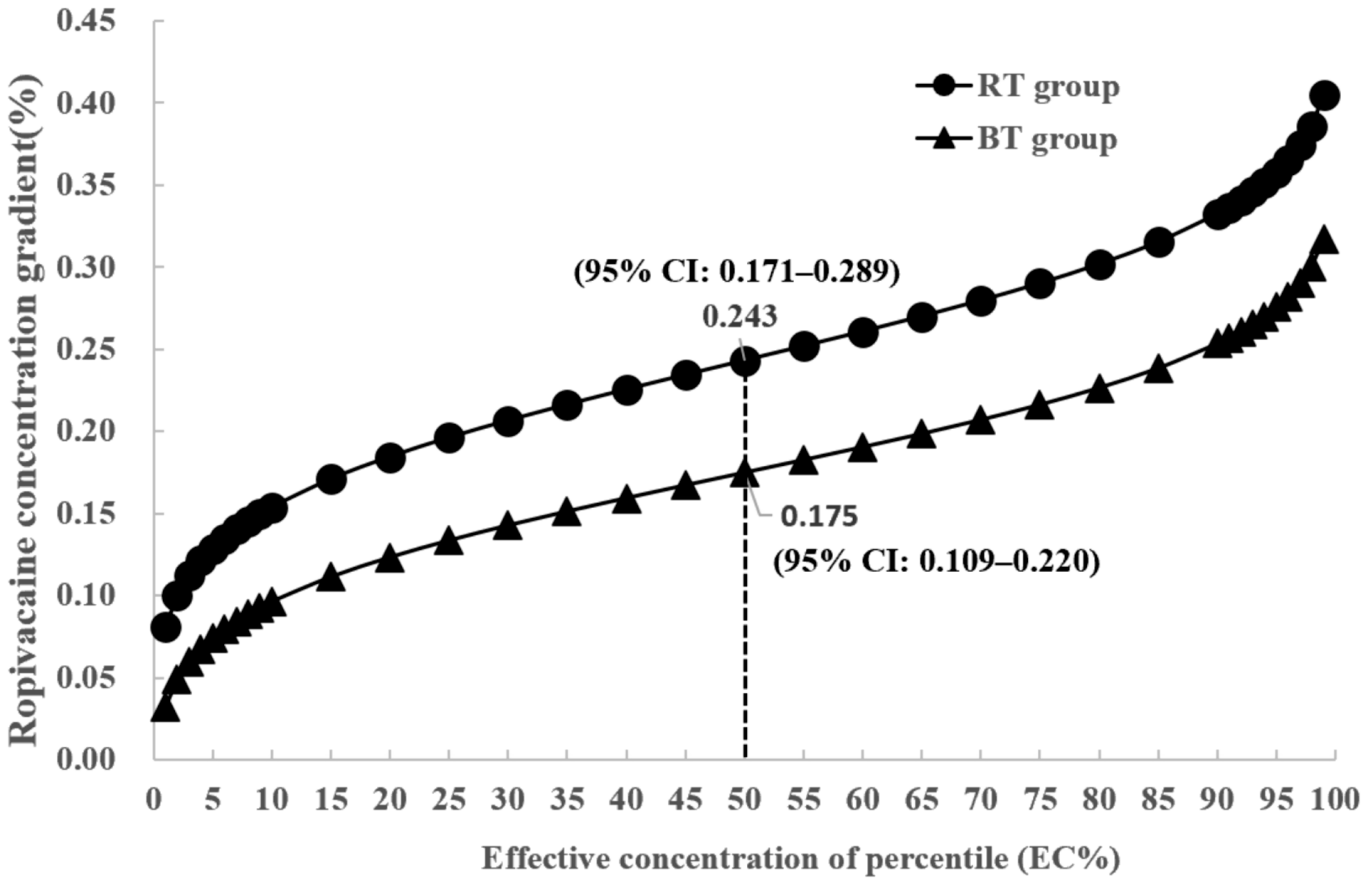
Concentration-effect analysis of ropivacaine on patients’ responses in the two groups.

Based on probit regression analysis, the EC_50_ of ropivacaine was significantly lower in the BT group (0.175, 95% CI: 0.109–0.220%) compared to the RT group (0.243, 95% CI: 0.171–0.289%) (*p* < 0.001), as illustrated in Figure 4.

### 3.5 Adverse events

There were no occurrences of local anesthetic toxicity or puncture-related complications in both groups.

## 4 Discussion

Our findings suggest that warming ropivacaine to 37°C reduced the concentration needed for effective nerve blockade, with significant decreases in media effective concentration for the BT group compared to the RT group. Specifically, the EC_50_ of ropivacaine in the BT group was 0.175% compared to 0.243% in the RT group.

Research has demonstrated that warming local anesthetics has potential advantages. It can reduce injection pain (18), accelerate the onset of nerve blocks, and improve block quality (19). However, the direct impact on nerve block efficacy remains controversial. Local anesthetics, being weak bases, are significantly influenced by their acid dissociation constant (pKa) (20). A lower pKa corresponds to a higher proportion of the non-ionized form, which enhances membrane permeability and anesthetic efficacy. Raising the temperature of local anesthetics reduces the pKa, which results in an increased fraction of non-ionized drugs (21). Others argue that while warming reduces the pain of injection and shortens onset time, it does not significantly enhance overall block success (18, 22). Our findings demonstrate that warming ropivacaine to 37°C significantly reduces the EC_50_ required for effective axillary brachial plexus block, suggesting that thermal modulation of local anesthetics may lower the minimum effective concentration necessary to achieve adequate anesthesia.

We observed a significantly longer median time to surgical readiness in the BT group (25 min) compared to the RT group (13 min), despite the potential kinetic advantages of warming. This finding may be explained by the significantly lower EC50 concentration used in the BT group, which might inherently require more time to achieve an effective neural blockade. Our study did not assess block duration, motor blockade due to the variability of ropivacaine concentrations among patients within each group. In this study, the effectiveness of the nerve block was primarily assessed based on sensory blockade, as it is the main determinant of surgical readiness. However, we acknowledge that motor blockade also plays a crucial role, particularly in surgeries requiring complete immobility of the operative limb (23). While sensory blockade ensures adequate analgesia, insufficient motor blockade could affect surgical conditions in certain procedures. In our study design, the concentration of ropivacaine was adjusted using the Dixon’s up-and-down method based on the success or failure of sensory nerve block. This led to a wide range of concentrations being used, which made it difficult to draw a reliable conclusion about the relationship between ropivacaine concentration and motor blockade. We also acknowledge that patients receiving lower concentrations of ropivacaine may experience reduced anesthetic efficacy and shorter durations of analgesia. This limitation underscores the importance of carefully weighing the risks and benefits associated with reducing ropivacaine concentration, particularly in longer or more complex surgeries. Further investigations are necessary to clarify the precise effects of warming local anesthetics on nerve block characteristics, particularly in terms of block duration and motor blockade. Future studies should also explore whether the benefits observed at 37°C extend to higher temperatures or whether excessive heating could compromise drug stability or increase the risk of nerve toxicity.

Research has shown that the concentration of local anesthetics is an important factor affecting both the onset and duration of nerve blocks (24). A 0.75% concentration of ropivacaine is frequently recommended for brachial plexus blocks in surgeries involving the upper limbs (6). Nonetheless, increasing the concentration of local anesthetic does not always lead to improved outcomes. Studies have indicated that 0.375% ropivacaine is non-inferior to 0.5% ropivacaine for achieving a successful ultrasound-guided brachial plexus block (25). Using lower concentrations of local anesthetics can achieve similar clinical efficacy while reducing the incidence of adverse events, making the selection of the effective concentration crucial. However, it is also known that reducing the concentration can decrease side effects but may shorten intraoperative and postoperative analgesia, making it less suitable for longer surgeries and lower concentrations of ropivacaine reduce toxicity risks, they may increase block failures, stressing the importance of balancing efficacy and safety. Fang et al. (26) found that the EC_90_ of ropivacaine was 0.257% at RT during supraclavicular brachial plexus block, comparable to the EC_50_ of 0.243% found in the present study. Although we also utilized 20 mL of ropivacaine, a potential reason for the difference in findings may be our use of a single-point injection technique for the axillary brachial plexus nerves, administering 5 mL of ropivacaine per nerve after ultrasound localization, as opposed to the single 20 mL injection used in the supraclavicular approach. The difference between the single - point injection technique used in this study for axillary brachial plexus nerves and the single 20 mL injection in the supraclavicular approach is significant, as the volume of local anesthetic plays a key role in determining the success of peripheral nerve blocks (24, 27, 28).

In the context of brachial plexus blocks, the median effective volume (EV_50_) of ropivacaine has been investigated in numerous studies. For instance, a study focusing on children aged 1 to 6 years found that the EV_50_ of 0.2% ropivacaine was 0.15 mL/kg at RT (29). This volume was critical for achieving effective anesthesia in pediatric patients undergoing upper extremity surgery. According to Song et al. (30) an effective volume of 17 mL is sufficient for achieving a successful ultrasound-guided supraclavicular brachial plexus block in 95% of patients. In contrast to the supraclavicular approach, the axillary brachial plexus is more anatomically dispersed, typically requiring a larger volume of local anesthetic, usually between 30 and 40 mL, to achieve adequate anesthesia. However, with ultrasound guidance, the required volume can be reduced (31). In our study, a total volume of 20 mL of ropivacaine was administered, with 5 mL injected around the radial, median, ulnar, and musculocutaneous nerves, respectively. Thus, the total volume of 20 mL used in this study is considered appropriate.

The limitations of this study can be summarized as follows: First, Dixon’s up-and-down method is a dose-finding methodology for peripheral nerve blocks (17). It can provide accurate results with a small sample size (15). This means that individual pain sensitivity differences may lead to inaccurate outcomes. For example, if a patient with a lower pain sensitivity has an ‘effective’ result at a certain concentration, the next patient may receive a lower concentration, which may not be effective for them, thus biasing the overall data. A larger sample size is needed to mitigate this issue and obtain more precise data. Second, while solutions were pre-warmed to 37°C, minor fluctuations during preparation or administration (e.g., syringe handling time) might have occurred, potentially affecting temperature consistency. Third, outcomes were assessed only during the intraoperative period. Long-term effects, such as postoperative neurologic complications or chronic pain outcomes, were not investigated. Fourth, although patients received dexmedetomidine for anxiolytic sedation, we did not measure sedation levels. Differences in the depth of sedation could influence patients’ subjective perception and reporting of sensory blockade during the assessment of block effectiveness at 30 min, potentially confounding the interpretation of block success.

In conclusion, our study determined the media effective concentrations of ropivacaine for axillary brachial plexus blocks at RT and BT. We found a statistically significant difference, with warmed ropivacaine requiring a lower concentration for adequate sensory blockade compared to RT. These findings support using warmed ropivacaine to enhance nerve block efficacy and reduce local anesthetic dosage.

## Data Availability

The raw data supporting the conclusions of this article will be made available by the authors, without undue reservation.
